# Software Tools to Facilitate Community-Based Surveillance: A Scoping Review

**DOI:** 10.9745/GHSP-D-22-00553

**Published:** 2023-10-30

**Authors:** Katherine Wallis, Vanessa Mwangale, Mikael Gebre-Mariam, Jenny Reid, Julia Jung

**Affiliations:** a Norwegian Red Cross, Oslo, Norway.; b Norwegian Red Cross, Nairobi, Kenya.

## Abstract

**Introduction:**

Public health surveillance traditionally occurs at a health facility; however, there is growing concern that this provides only partial and untimely health information. Community-based surveillance (CBS) enables early warning and the mobilization of early intervention and response to disease outbreaks. CBS is a method of surveillance that can monitor a wide range of information directly from community members. CBS can be done using short message service, phone calls, paper forms, or a specialized software tool. No scoping review of the available software tools with the capability for CBS exists in the literature. This review aims to map software tools that can be used for CBS in both community health programs and emergency settings and demonstrate their use cases.

**Methods:**

We conducted a scoping review of academic literature and supplemental resources and conducted qualitative interviews with stakeholders working with digital community health and surveillance tools.

**Results:**

All of the tools reviewed have features necessary to support the reporting process of CBS; only 3 (CommCare, Community Health Toolkit, and DHIS2 Tracker) provided all 10 attributes included in the mapping. AVADAR and Nyss were the only tools designed specifically for CBS and for use by volunteers, while the other tools were designed for community health workers and have a broader use case.

**Conclusion:**

The findings demonstrate that several software tools are available to facilitate public health surveillance at the community level. In the future, emphasis should be put on contextualizing these tools to meet a country's public health needs and promoting institutionalization and ownership by the national health system. There is also an opportunity to explore improvements in event-based surveillance at the community level.

## Abstract (precis)

Authors of this review identified software tools that could be used for community-based public health surveillance and facilitate faster communication of potential outbreaks, reporting, and coordination of response efforts.

## Teaser

**Key Findings:**

- Of the 8 software tools mapped in this scoping review, only 2 were specifically designed for community-based public health surveillance (CBS) and to be used by volunteers.
- All the tools had features that could support CBS, but only 3 tools had all 10 attributes: freely available source code, feature and smartphone ready, web and mobile ready, automated dashboard, case management documentation and tracking, text message reporting capability, offline capability, data entry guided assessment, geolocation capability, and integration with other systems.
- A current gap in most of the CBS software tools is event-based surveillance, which could provide information from community members.

**Key Implications:**

- There is a need for better coordination and alignment of development actors with local ministries to avoid fragmented and parallel tools.
- Sufficient emphasis should be placed on extending existing digital tools to CBS and institutionalizing these functions among local stakeholders rather than incorporating third-party tools for CBS in environments with preexisting Integrated Disease Surveillance and Response tools, which, despite being integrated, can result in duplication and increased administrative and technical burden.

## INTRODUCTION

Public health surveillance is “the continuous and systematic collection, orderly consolidation and evaluation of pertinent data with prompt dissemination of results to those who need to know” and an essential part of a national health system.^1^ Public health surveillance traditionally occurs at a health facility. Information is collected from primary and secondary health facilities and fed into the national surveillance system.^2^ These public health surveillance systems can be delayed and fragmented,^3^ and there is growing concern that this provides only partial and untimely health information.^4^^,^^5^ Information and potential cases of infectious diseases can be missed, either because not all individuals visit the health care facilities when they fall sick^4^^,^^5^ due to an inability to reach a health facility^2^ or because reporting at the health facility is not functional (e.g., due to conflict, disaster, or protracted crisis). If a developed system is not in place to systematically perform public health surveillance, this can be a barrier to public health interventions at the country level.^6^ Community-based surveillance (CBS) can fill this gap in surveillance by leveraging community members to identify and report significant public health events.^7^^,^^8^

CBS enables early warning and the mobilization of early intervention and response to disease outbreaks.^9^^–^^14^ CBS is a method of surveillance that can monitor a wide range of information directly from community members.^2^ It is a simple, adaptable, and low-cost public health methodology that is facilitated by the communities it works to protect.^2^ Two factors distinguish CBS from other surveillance systems: (1) the detection of the event occurs outside the health facility and (2) the individuals who detect these incidences are community members.^7^ Involving community members in the detection and reporting of public health events can help to overcome some of the limitations in public health surveillance.^15^ A community in this article refers to a group of persons living within a geographical location; for each CBS project, the size of this community may vary greatly.

Involving community members in the detection and reporting of public health events can help to overcome some of the limitations in public health surveillance.

In a CBS program, preselected and trained community volunteers or community health workers identify and report predetermined health risks by using community case definitions and unusual events.^8^ A community case definition is a broad description of signs and symptoms used to identify the predetermined disease.^12^ Community volunteers or community health workers identify sick people in their community and check to see if the community case definition matches.^8^ In doing so, they can help identify diseases (e.g., measles, cholera, or poliomyelitis) and alert health authorities.^8^

By reporting on an unusual event, such as multiple animal deaths or a health risk, community members can alert health authorities at the early stages of an outbreak^8^ to help prevent spread. Reports from the community are sent to a health care worker at a health delivery point or assigned supervisors within a community health program (e.g., staff from a health authority).^8^ They then cross-check the report from the community to see if it matches the respective community case definition. If it matches, they escalate the alert to the health authorities at the district level—and in some cases, also directly to the state or regional levels—for further investigation to confirm the disease and facilitate early response. CBS is a method of Integrated Disease Surveillance and Response (IDSR), an approach that uses standardized tools to collect data for multiple diseases, ensuring early warning and quick response.^8^ Electronic IDSR (eIDSR) applies digital tools to the IDSR approach.^16^

The method of reporting by community members varies depending on the context. Reports can be sent using short message service (SMS), phone calls, paper forms, or a specialized software tool. The use of a software tool for CBS, rather than paper, SMS, or phone calls, allows for faster sending of reports on potential outbreaks. Depending on the tool, it can also provide recordkeeping of reports, analysis, and response or interventions, promoting communication between actors and ministries of health. Some of the software tools that can be used for CBS are designed specifically for that purpose, while others are designed to be used for a variety of community- or facility-level activities with the capability to be used for CBS.

CBS is an epidemic preparedness and emergency response methodology that can be used as a component of a comprehensive community health program. Although there are several software tools to facilitate CBS, no current scoping review of available software tools exists. This review aims to map the software tools that can be used for CBS in both community health programs and emergency settings and demonstrate their use cases.

## METHODS

We conducted a scoping review of academic literature and supplemental resources of software tools. Qualitative interviews with stakeholders working with community health digital tools were used to gain supplemental information and insight into experiences on using the tools.

### Literature Review

The literature review focused on articles that described a community surveillance methodology program that used a software tool to facilitate surveillance. We included those articles about low- and middle-income country settings. We searched PubMed for articles published in English between January 2010 and March 2023. The key terms used for the literature review included “community-based surveillance,” “community surveillance,” “event-based surveillance,” “public health surveillance,” “epidemiological surveillance,” “eIDSR,” and “electronic integrated disease surveillance and response.” The Google search engine was used to obtain supplemental information about each tool.

We used the literature review to select software tools for inclusion in the mapping. Due to the sheer volume of disease surveillance and response digital tools available, the tools that were included in this review needed to have a feature that could be used for CBS. Not all tools included are actively being used for CBS, but all have been designed with a feature that could enable their use for CBS. The literature was reviewed, and an analysis of their features was developed.

To complete this analysis or “mapping,” we used additional resources, including user manuals and overview documents for the software platforms, websites for the tools, and PowerPoint presentations.

### Qualitative Interviews

To provide additional information for the mapping, we conducted 20 qualitative interviews with stakeholders working with digital community health and surveillance tools. The participants worked either in the development of the tools or with the implementation of the tools in community health or emergency response programming. Sampling was purposive with the use of gatekeepers. The tools covered through interviews included KoboToolbox; Early Warning, Alert and Response System (EWARS); DHIS2 Tracker; Surveillance Outbreak Response Management and Analysis System (SORMAS); Community Health Toolkit; and Nyss. The authors were unable to hold an interview with anyone who had experience using Auto-Visual Acute Flaccid Paralysis Detection and Reporting (AVADAR) or CommCare.

#### Data Collection

All but 1 interview took place digitally, with the use of an interview guide. The aim of the review was explained at the beginning of each interview, and participants gave their verbal consent. We did not seek ethical approval to conduct the interviews. The first 3 interviews occurred with 2 authors present; the remaining took place with 1 author. When interviewees granted permission, the interviews were audio-recorded, then transcribed and reviewed to ensure accuracy.

#### Data Analysis

The analysis methodology was thematic analysis, as described by Guest et al. in *Applied Thematic Analysis.*^17^ Thematic analysis was chosen due to its inductive nature and because it is designed to examine themes in a way that is both transparent and credible. After the interviews were transcribed, the transcripts were coded, and themes were generated based on the codes. After completion of the analysis, discussion and comparison took place until reviewers agreed with the final themes.

## RESULTS

A total of 4,787 articles were reviewed for inclusion, and 71 full-text studies were then assessed for full eligibility. After excluding 55 studies, 16 articles were selected for review (Supplement).^18^^–^^36^ We used the Preferred Reporting Items for Systematic reviews and Meta-Analyses flow diagram to outline the screening process (Figure).^37^ During review, an additional 3 articles found from the reference lists of the articles were also included. We chose which tools to include in the mapping based on the literature review, with the exception of 2 additional tools that were found based on the expertise of the authors; we found no academic literature that referred to them. Eight tools were initially selected to be included in the mapping based on the eligibility criteria (Table 1). The capabilities and attributes of the tools, as highlighted in the literature review, formed the foundation of the mapping (Table 2). Initially, we identified 8 attributes of these tools to include in the mapping, 2 of which—phone requirements and device readiness—interviewees confirmed as being important. Interviewees also highlighted the need to include 2 additional attributes, integration with other applications and offline capabilities, so those attributes were added to the mapping.

**TABLE 1.** Main Use of Software Tools for Facilitating Community-Based Surveillance

Tool Name and Organization	Main Use
Auto-Visual Acute Flaccid Paralysis Detection And Reporting (AVADAR), eHealth Africa	Detection and reporting of acute flaccid paralysis (which may indicate poliomyelitis) in communities by community members. Volunteers or community health workers send a report if they see a child presenting with acute flaccid paralysis.
Commcare, Dimagi	For community health workers or health workers to receive prompts for health activities and collect data on these activities.
Community Health Toolkit, Medic Mobile	For community health workers or health workers to receive prompts for health activities and collect data on these activities.
DHIS2 Tracker, University of Oslo	Individual-level data collection and reporting at community or health facility level.
Early Warning, Alert and Response System (EWARS), World Health Organization	Emergency settings to facilitate early detection and response to outbreaks at health facility or community level.
KoboToolbox, Kobo	Data collection and assessment tasks.
Nyss, Norwegian Red Cross	Detection and reporting of health risks in communities by community members.
Surveillance Outbreak Response Management and Analysis System (SORMAS), Helmholz	Disease control and outbreak detection in an emergency at health facility, point of entry, or community.

**TABLE 2.** Attribute Key for Software Tools for Facilitating Community-Based Surveillance

Attribute	Attribute Description
Source code	Open or closed: The source code is freely available.
Phone requirements	Feature phone and smartphone. A feature phone does not usually support add-on applications. It may also have limited processing and storage capacity and lack advanced multimedia and Internet connectivity options available on smartphones.
Device readiness	Web and mobile ready: Application is available for both mobile device and computer.
Dashboard	The tool has automated dashboard capability.
Case management	The tool can, to some extent, document and track follow-up on cases.
Integration with other apps	Possesses the ability to connect to other applications, such as national level electronic integrated disease surveillance and response systems.
Offline capability	Can be used offline. The application updates to the database once Internet connectivity is available.
Guided assessment	The tool has a digital form, which guides a user through the data entry process. It facilitates the user to do an assessment of the clinical case.
Geolocation capability	Ability to display tagged geolocation.
SMS reporting capability	Reports can be sent through SMS directly into the tool.

Abbreviation: SMS, short message service.

**FIGURE.**
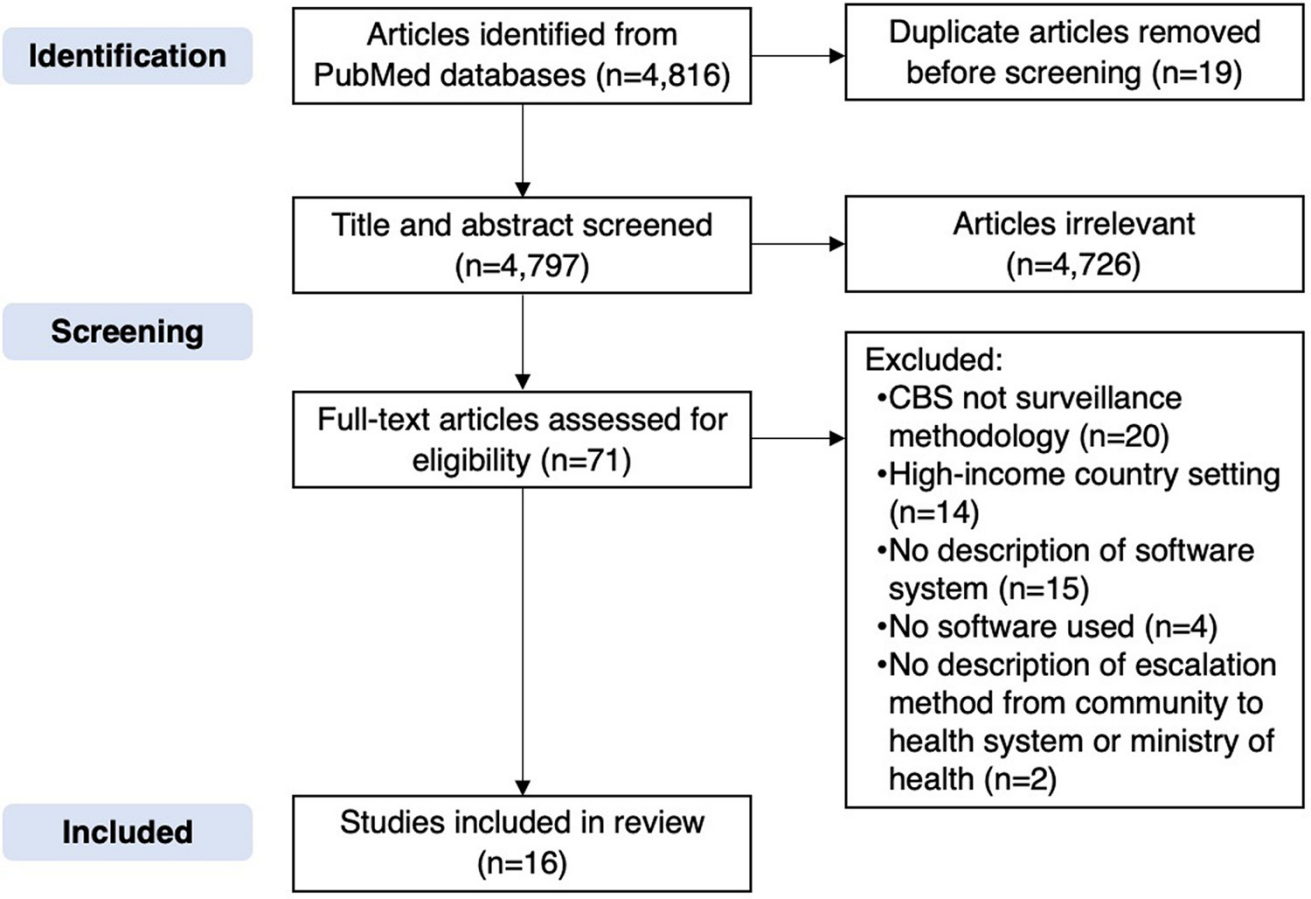
PRISMA Flow Diagram for Selecting Studies on Software Tools to Facilitate Community-Based Surveillance Abbreviations: CBS, community-based surveillance; PRISMA, Preferred Reporting Items for Systematic reviews and Meta-Analyses.

### Comparison of Software Tools

The mapping demonstrates the individual software tools available for CBS and provides a comparison of their features (Table 3). Some tools have been designed specifically for CBS, whereas other tools have been designed for an alternative use but have features that enable them to also be used for CBS. The tools AVADAR and Nyss have been designed specifically for CBS and for use by volunteers, while the other tools were designed for use by community health workers or health care workers and have a broader use case. One tool, EWARS, was designed for use both in health facilities and communities^38^; however, the review found no examples of its use at the community level by community health workers or volunteers. SORMAS has been designed for data collection at health facilities, entry points, and communities,^39^ but no examples of data entry occurring at the community level were found.

**TABLE 3.** Mapping of Software Tools to Facilitate Community-Based Surveillance

	Open Source Code	Web and Mobile Ready	Feature and Smartphone	Dashboard	Case Management Ability	Integration With Other Apps	Offline Capability	Guided Assessment	Geolocation Capability	SMS Reporting Capability
**AVADAR**	Closed	Yes	Smart	Yes	No	No	No	Yes	Yes	No
**CommCare**	Yes	Yes	Yes	Yes	Yes	Yes	Yes	Yes	Yes	Yes
**Community Health Toolkit**	Yes	Yes	Yes	Yes	Yes	Yes	Yes	Yes	Yes	Yes
**DHIS2 Tracker**	Yes	Yes^a^	Yes	Yes	Yes	Yes	Yes	Yes	Yes	Yes
**EWARS**	Yes	Yes	Smart^b^	Yes	Yes	No	Yes	Yes	Yes	No
**KOBO Toolbox**	Yes	Yes	Smart^b^	Yes	No	Yes	Yes	Yes	Yes	No
**Nyss**	Yes	Web only^b^	Yes	Yes	No	No	No	No	Yes	Yes
**SORMAS**	Yes	Yes	Smart^b^	Yes	Yes	Yes	Yes	Yes	Yes	No

Abbreviations: AVADAR, Auto-Visual Acute Flaccid Paralysis Detection and Reporting; EWARS, Early Warning, Alert and Response System; SMS, short message service; SORMAS, Surveillance Outbreak Response Management and Analysis System.

a DHIS2 Tracker has a mobile-based application through DHIS2 Capture.

b Attribute partially present in software tool.

The tools CommCare, Community Health Toolkit, DHIS2 Tracker, and Nyss have SMS capabilities, allowing data entry directly into the tool via SMS.^15^^,^^40^^,^^41^ The mode of data entry for AVADAR, KoboToolbox, and SORMAS is a form on an app. CommCare, Community Health Toolkit, and DHIS2 Tracker can accommodate data entry through a form or SMS, depending on the program's needs. A form can function as a guided assessment for community health workers to assist with decision-making in clinical case management.

CommCare, Community Health Toolkit, DHIS2 Tracker, and Nyss offer a function to enable data entry on a feature phone.^15^^,^^40^^,^^42^ CommCare, Community Health Toolkit, DHIS2 Tracker, EWARS, and SORMAS allow offline use.^39^^,^^43^^–^^46^ This function enables data entry into a form when offline; the data uploads to the server once the device is connected to the Internet.

All the software tools are available in different languages. Nyss can be used in English, French, Spanish, or Arabic, and the Community Health Toolkit is available in English, French, Hindi, Nepali, Spanish, Swahili, and Indonesian.^47^ CommCare is functional in English, Spanish, Portuguese, and French^48^; EWARS is available in French, English, and Arabic^49^; and AVADAR is available in 17 languages.^50^ A language translation feature in SORMAS allows for it to be changed into any language.^35^ DHIS2 is currently available in English, French, Spanish, Portuguese, Hindi, Vietnamese, Chinese, and Norwegian. The interface and metadata can also be translated into additional languages.^51^

### Stakeholder Experiences With Using Software Tools for CBS

The interviews aimed to gain an understanding of the experiences of those using software platforms for CBS projects and their perceptions of these software tools. The quotes in this section are from those involved in implementation of CBS, either directly or at a strategic level.

#### Key Characteristics of an Effective Community-Based Software Tool

A common belief among interviewees was that a CBS system should be simple and user-friendly, enabling community members with varying levels of education to perform CBS.

A common belief among interviewees was that a CBS system should be simple and user-friendly, enabling community members with varying levels of education to perform CBS.

*You need to have a tool that is simple, that they can easily grasp it shouldn't be complicated.* —Ministry of Health representative

*The other issue that I will comment is, I think the tool should be user friendly. If it is complicated, it becomes difficult.* —Ministry of Health representative

Offline capability was also emphasized, with the suggestion that a tool should not be dependent on the Internet. Another key desire in a tool was automation, including the ability to generate automated reports to the ministry of health, reports to volunteers, and a summary of alerts. Tool contextualization was seen as an important characteristic for a CBS tool, meaning it should work in existing contexts and be adaptable.

*Make it practical as possible. Secondly, it's has to be adaptable to the context.* Humanitarian organization representative

Integration into broader digital health systems was mentioned by a number of interviewees, and this was closely connected to the sustainability of a system.

*Because if you want to make surveillance, you have to match it to the existing system, otherwise, it won't work after the project.* —Humanitarian organization representative

*Then another factor that we need to consider is the issue of interoperability, that, you know these systems should be talking to each other.* —Ministry of Health representative

The support and facilitation of rapid response, including through the sharing of information in real time, was also deemed important.

*The mission is to detect it [CBS] early, so that we can respond as soon as possible.* —Humanitarian organization representative

*Because for community-based surveillance, it has to be a real-time surveillance.* —Humanitarian organization representative

#### Challenges With CBS Projects Using a Software Tool

Interviewees noted technical challenges with some CBS tools, including the need for Internet connectivity for up to 24 hours and data linkage, referring to the connection of the data collected in 1 system with other systems being used in the country.

Interviewees also highlighted that phones could cause difficulties, with some community members not having a phone or only having a simple feature phone. The challenges with access to phones could also be connected to women not having access to phones, as described by an interviewee speaking about community health volunteers.

*So most probably it's a female, female or mother and somebody who is married, and in the community or who lives in the community and doesn't go anywhere else. They don't really have phones. These women today. They have a simple button phone, or they share phones with their husband.* —Humanitarian organization representative

An interviewee articulated that providing electronic equipment to start a CBS project is not the answer to this.

*You find that when partners come … they (NGOs) might give them gadgets, so you find that some [Health Surveillance Assistants] have 2, 3 gadgets or funds and while others they don't have any… I've said so why not check for 1 gadget … because each donor wants to identify, itself, always a particular tools or infrastructure, so it becomes difficult.* —Ministry of Health representative

Finally, power and electricity to charge phones was a point of concern in the success of a CBS project.

*So power is also a challenge… unless you have alternative source of power. For you to charge you know, the equipment.* —Ministry of Health representative

## DISCUSSION

The results of our review indicate redundancies in several software tools designed to support community-level public health surveillance. Although all the tools reviewed had features necessary to support the reporting process of CBS, only 3 (CommCare, Community Health Toolkit, and DHIS2 Tracker) provided all 10 attributes included in the mapping. The attributes can be considered by implementers when selecting a tool for CBS, based on their importance for the effectiveness and sustainability of the specific program. Other factors, such as cost and any preexisting use of a tool in the country, should also be considered in tool selection.

Given that early reporting on public health risks is a key purpose of CBS, tools that offer various data entry methods may be more adaptable to varying local requirements. For example, interviewees discussed how data entry through SMS has broader utility among users with limited digital literacy and users in remote locations. However, the most common mode of data entry is through mobile or web applications, which require Internet connectivity. This may pose a challenge in some contexts where there is limited connectivity or a significant fee for connectivity. On the other hand, mobile and web applications can offer the user more features, such as an intuitive user interface and a guided assessment to help direct reporting and decision-making. This may improve reporting accuracy, as it can prompt the user during their assessment and prompt for follow-up activities. The tools CommCare, Community Health Toolkit, and DHIS2 Tracker offer data entry through both SMS and a mobile form, which may make them good options when multiple data entry methods would be beneficial.

Tools that offer various data entry methods, including both SMS and Internet-based, may be more adaptable to varying local requirements.

Over the past 2 decades, the proliferation and effectiveness of small-scale and siloed digital health applications has been an area of concern in many countries.^52^^,^^53^ However, IDSR places a strong emphasis on integration, not only in data collection forms, standards, and case definitions but also through the harmonization of digital tools to facilitate a coordinated surveillance and response process. As the tools included in our mapping showcase, tools not developed specifically for CBS still have the capability to facilitate it. These tools may be a better option for implementers looking to select a tool with the intention of branching out beyond CBS in the future. A better option would be to focus on taking tools already in use by national health systems and expanding their capabilities to enable CBS.

A prevailing agreement is emerging that countries should transition toward a more unified strategy when implementing IDSR tools. This involves not only aligning software but also data collection forms, communication channels, and case definitions to maximize efforts among disease surveillance and response programs and stakeholders. This will facilitate coordinated public health surveillance in both emergencies and community health programs and prevent parallel digital systems.^54^ Interview participants highlighted this as an important factor. To achieve this aim, numerous ministries of health in the Global South have initiated national digital health strategies and governance procedures. Enhancing these processes will prove pivotal as digital tools become more prevalent and funding for their implementation grows.

In recent years, there has also been a growing emphasis on digital tools that are not only configurable to fit the needs of a variety of contexts but can also accommodate new features and use cases.^53^ DHIS2 is an example of a tool that has catered to this demand. DHIS2 has a configurable data model that allows adaptation according to changing needs. It also has an AppHub, which allows third parties, such as the CBS implementing team in a specific context, to develop custom web and Android apps as an extension to the core application. Community Health Toolkit also has been developed to be configurable for different use cases in community health.^45^ Using a core framework provides a foundation for app development that can be altered according to the needs of the program. This style of software tool may also lead to increasing capacity by the implementing team as they work to develop the application to meet the specific needs of a given context.

In some cases, surveillance may be improved by adopting a tool specifically designed for CBS. For instance, the Nyss software tool, used by Red Cross Red Crescent community volunteers, offers the added value of introducing surveillance officers into the national disease surveillance workflow. Community volunteers send alerts when someone is unwell in their community based on health risks and according to predefined community case definitions. In 1 country, the integration of Nyss with the national Ministry of Health eIDSR system is currently being explored. The integration of Nyss into eIDSR means community-level data can be entered directly into a national health information system rather than in a separate parallel information system.

Despite the benefits offered by CBS-specific tools, such as Nyss, the silo-based approach is becoming increasingly less common in favor of tools that offer a broader use case. Indicative of this, CommCare, Community Health Toolkit, DHIS2 Tracker, and KoboToolbox have features that cater to broader community health or health care facility activities. The tools EWARS and SORMAS do not cater to broadened community health activities but do cater to surveillance from a variety of settings. However, it should be noted that not all features of these tools are often simultaneously operational in the contexts where they are deployed. This scoping review has not explored the different costs for each tool, including development and implementation.

In the future, broader organizational factors will also influence the ongoing development of the software tools available for CBS. Scaling the number of registered and active users to where CBS coverage is adequate for a specific population size requires ongoing training and community engagement; therefore, tools should be designed to be user-friendly and intuitive. In some cases, tools may need to be used by community members with a variety of education and digital literacy levels. Similarly, the linkage of health facilities and district offices to CBS is critical to ensure adequate supervision and follow-up. More broadly, the scale of adoption and extent to which a CBS tool becomes integrated into a national government health system are factors that can influence the scalability of any tool.

The development of new tools should not be a priority. This scoping review demonstrates that a variety of tools exist to facilitate CBS. Rather, emphasis should be placed on contextualizing these tools to meet the public health needs of countries and linking them with the existing eIDSR systems to prevent parallel systems. In addition, national ownership should be the goal of all programs using tools for CBS.

Emphasis should be placed on contextualizing existing tools to meet the public health needs of countries and linking them with the existing eIDSR systems to prevent parallel systems.

Looking ahead, a critical area where most of the tools fall short is also an area of CBS that is the weakest and most complex. Currently, significant gaps exist in community event-based surveillance (CEBS). Unlike indicator-based surveillance, CEBS is a component of CBS that involves the reporting of unusual events not only by designated community focal points but community members at large,^8^ giving an opportunity to receive information from a broader level without having to train a workforce. Reported events can include information that may be unconfirmed, incomplete, or simply rumors, such as a cluster of animal deaths. Such public-facing systems that support CBS at a large scale are rare. Although some tools, specifically Nyss, EWARS, and DHIS2, offer a feature for event-based surveillance, an opportunity still exists for development to increase the scalability of CEBS. Further efforts are needed to explore how digital tools can support the core functions of CEBS, primary of which is the analysis and interpretation of unstructured data. The capability of CBS tools to parse unstructured text, primarily reported by the public, is no longer implausible given the recent advancements in text analysis powered by artificial intelligence. Currently, CEBS implementations that receive reports through hotlines or free-text SMS, such as SORMAS and EWARS, rely on a labor-intensive manual review process.

While both event- and indicator-based surveillance strategies focus on collecting community-level information, the type of information collected, who reports it, and how it is processed have implications not only on the features and functionality of the digital solution but also on the technical human resources needed to facilitate this process at scale. The 2 components of CBS must also be integrated to ensure cross-verification between event and indicator data at the community level.

### Limitations

It was beyond the scope of this review to map all software tools used in public health surveillance. The scoping review prioritized those that met the eligibility criteria and those that had the capability to facilitate CBS. Although we tried to map all current features of the included software tools, we recognize that ongoing developments may not have been captured. Additionally, it was outside of the scope of this review to include details about the costs associated with each software tool, but this could be explored in the future. Cost, including for setup and long-term implementation, should always be considered as part of planning a CBS implementation.

## CONCLUSION

The findings demonstrate that several software tools are available to facilitate CBS, but only 3 tools had all 10 attributes included in the mapping. In the future, emphasis should be on coordinating and alignment between development actors with the local ministries. Existing tools should be extended and institutionalized, rather than introducing new tools. Event-based surveillance is an area that has not been sufficiently developed.

## Supplementary Material

GHSP-D-22-00553-supplement.pdf

## Notes

**Peer Reviewed**

**First published online:** October 3, 2023.
