# Supplementary material for: Software Tools to Facilitate Community-Based Surveillance: A Scoping Review
Source: Glob Health Sci Pract. 2023 Oct 30;11(5):e2200553. doi: 10.9745/GHSP-D-22-00553 (PMC10615241; doi:10.9745/GHSP-D-22-00553)
Supplement: GHSP-D-22-00553-supplement.pdf [file GHSP-D-22-00553-supplement.pdf]

Supplement to: Wallis K, Mwangale V, Gebre-Mariam M, Reid J, Jung J. Software tools to facilitate community-based surveillance: a scoping review. *Glob Health Sci Pract*. 2023;11(5):e2200553. https://doi.org/10.9745/GHSP-D-22-00553

SUPPLEMENT. ARTICLES INCLUDED IN THE SCOPING REVIEW OF SOFTWARE TOOLS TO FACILITATE COMMUNITY-BASED SURVEILLANCE

Title	Software Tool
Silenou BC, Nyirenda JLZ, Zaghloul A, et al. Availability and suitability of digital health tools in Africa for pandemic control: scoping review and cluster analysis. *JMIR Public Health Surveill*. 2021;7(12):e30106. CrossRef. Medline	AVADAR<br>CommCare<br>DHIS2<br>Kobo ToolBox<br>EWARS<br>SORMAS
Ticha JM, Akpan GU, Paige LMF, et al. Outcomes of the deployment of the Auto-Visual Acute Flaccid Paralysis Detection and Reporting (AVADAR) system for strengthening polio surveillance in Africa from 2017 to 2018: evaluation study. *JMIR Public Health Surveill*. 2020;6(4):e18950. CrossRef. Medline	AVADAR
Shuaib FMB, Musa PF, Gashu ST, et al. AVADAR (Auto-Visual AFP Detection and Reporting): demonstration of a novel SMS-based smartphone application to improve acute flaccid paralysis (AFP) surveillance in Nigeria. *BMC Public Health*. 2018;18(Suppl 4):1305. CrossRef. Medline	AVADAR
Diallo M, Traore A, Nzioki MM, et al. Auto Visual AFP Detection and Response (AVADAR) improved polio surveillance in Lake Chad polio outbreak priority districts. *J Immunol Sci*. 2021;Spec Issue(2):1101. CrossRef. Medline	AVADAR
Dayalu R, Wacksman J, Chen M, Loudon M, Lesh N. Adoption and continued use of mobile technology: an analysis of CommCare data. *Procedia Engineering*. 2015;107:247–254. CrossRef	CommCare
MacDonald ME, Diallo GS. Socio-cultural contextual factors that contribute to the uptake of a mobile health intervention to enhance maternal health care in rural Senegal. *Reprod Health*. 2019;16(1):141. CrossRef. Medline	CommCare
Biemba G, Chiluba B, Yeboah-Antwi K, et al. A mobile-based community health management information system for community health workers and their supervisors in 2 districts of Zambia. *Glob Health Sci Pract*. 2017;5(3):486–494. CrossRef. Medline	DHIS2
Reynolds E, Martel LD, Bah MO, et al. Implementation of DHIS2 for disease surveillance in Guinea: 2015-2020. *Front Public Health*. 2022;9:761196. CrossRef. Medline	DHIS2
Joseph Wu TS, Kagoli M, Kaasbøll JJ, Bjune GA. Integrated Disease Surveillance and Response (IDSR) in Malawi: Implementation gaps and challenges for timely alert. *PLoS One*. 2018;13(11):e0200858. CrossRef. Medline	DHIS2

Supplement to: Wallis K, Mwangale V, Gebre-Mariam M, Reid J, Jung J. Software tools to facilitate community-based surveillance: a scoping review. *Glob Health Sci Pract*. 2023;11(5):e2200553. https://doi.org/10.9745/GHSP-D-22-00553

Title	Software Tool
Karo B, Haskew C, Khan AS, Polonsky JA, Mazhar MKA, Buddha N. World Health Organization Early Warning, Alert and Response System in the Rohingya Crisis, Bangladesh, 2017–2018. *Emerg Infect Dis*. 2018;24(11):2074–2076. CrossRef. Medline	EWARS
Sheel M, Collins J, Kama M, et al. Evaluation of the early warning, alert and response system after Cyclone Winston, Fiji, 2016. *Bull World Health Organ*. 2019;97(3):178–189C. CrossRef. Medline	EWARS
Muscatello DJ, Manurung MK, Reo SEN, Pardosi JF. Evaluation of the Indonesian Early Warning Alert and Response System (EWARS) in West Papua, Indonesia. *WHO South-East Asia J Public Health*. 2020;9(2):111–117. CrossRef. Medline	EWARS
Benitez-Valladares D, Kroeger A, Tejeda GS, Hussain-Alkhateeb L. Validation of the Early Warning and Response System (EWARS) for dengue outbreaks: evidence from the national vector control program in Mexico. *PLoS Negl Trop Dis*. 2021;15(12):e0009261. CrossRef. Medline	EWARS
Cardenas R, Hussain-Alkhateeb L, Benitez-Valladares D, Sánchez-Tejeda G, Kroeger A. The Early Warning and Response System (EWARS-TDR) for dengue outbreaks: can it also be applied to chikungunya and Zika outbreak warning? *BMC Infect Dis*. 2022;22(1):235. CrossRef. Medline	EWARS
Hussain-Alkhateeb L, Kroeger A, Olliaro P, et al. Early warning and response system (EWARS) for dengue outbreaks: recent advancements towards widespread applications in critical settings. *PLoS One*. 2018;13(5):e0196811. CrossRef. Medline	EWARS
Byrne A, Nichol B. A community-centred approach to global health security: implementation experience of community-based surveillance (CBS) for epidemic preparedness. *Glob Secur Health Sci Policy*. 2020;5(1):71–84. CrossRef	Kobo Toolbox
Silenou BC, Tom-Aba D, Adeoye O, et al. Use of Surveillance Outbreak Response Management and Analysis System for human monkeypox outbreak, Nigeria, 2017-2019. *Emerg Infect Dis*. 2020;26(2):345–349. CrossRef. Medline	SORMAS
Tom-Aba D, Silenou BC, Doerrbecker J, et al. The Surveillance Outbreak Response Management and Analysis System (SORMAS): Digital Health Global Goods Maturity Assessment. *JMIR Public Health Surveill*. 2020;6(2):e15860. CrossRef. Medline	SORMAS
Fähnrich C, Denecke K, Adeoye OO, et al. Surveillance and Outbreak Response Management System (SORMAS) to support the control of the Ebola virus disease outbreak in West Africa. *Euro Surveill*. 2015;20(12):21071. CrossRef. Medline	SORMAS
